# Developing strategies to address barriers for tuberculosis case finding and retention in care among refugees in slums in Kampala, Uganda: a qualitative study using the COM-B model

**DOI:** 10.1186/s12879-022-07283-9

**Published:** 2022-03-28

**Authors:** Esther Buregyeya, Edwinah Atusingwize, Juliet N. Sekandi, Richard Mugambe, Rebecca Nuwematsiko, Lynn Atuyambe

**Affiliations:** 1 Department of Disease Control and Environmental Health, School of Public Health, College of Health Sciences, Makerere University, Kampala, Uganda; 2 Department of Epidemiology and Biostatistics, College of Public Health, University of Georgia, Athens, GA USA; 3 Department of Community Health and Behavioural Sciences, School of Public Health, College of Health Sciences, Makerere University, Kampala, Uganda

**Keywords:** Refugees, TB case finding and retention in care, COM-B model, Behaviour change wheel

## Abstract

**Background:**

Globally, displaced populations face an increased burden of tuberculosis (TB). Uganda is currently hosting unprecedented big numbers of refugees from the East African region. Recent evidence shows increased spread of multi-drug resistant TB (MDR-TB) across East Africa as a result of migrants from Somalia- a high MDR-TB prevalent country, calling for urgent identification and management of cases for the countries in the region. One of the strategies recommended is optimization of diagnosis, treatment and prevention of TB in refugees. This study aimed at exploring the barriers to and facilitators for TB case finding and retention in care among urban slum refugees and suggestions on how to improve. This was to guide the development of interventions to improve TB case finding and retention in care among the said population.

**Methods:**

A cross-sectional study utilizing qualitative methods was conducted among refugees in an urban slum in Kampala City, Uganda. Key informant interviews with health care workers and community leaders and in-depths interviews with refugee TB patients and care takers of TB patients were conducted (30 interviews in total). Interview questions were based on constructs from the COMB-B model (Capability, Opportunity and Motivation Model of Behaviour change). Manual content analysis was performed and identified targeted intervention strategies guided by the related Behavior Change Wheel implementation framework.

**Results:**

Key barriers included; physical capability (availability of and easily accessible private facilities in the community with no capacity to diagnose and treat TB), psychological capability (lack of knowledge about TB among refugees), social opportunity (wide spread TB stigma and language barrier), physical opportunity (poor living conditions, mobility of refugees), reflective motivation (lack of facilitation for health workers), automatic motivation (discrimination and rejection of TB patients). Facilitators were; physical capability (availability of free TB services in the public health facilities), social opportunity (availability of translators). We identified education, incentivization, training, enablement, and restructuring of the service environment as relevant intervention functions with potential to address barriers to and enhance facilitators of TB case finding and retention among refugees in urban slums.

**Conclusion:**

The key barriers to TB control among refugees living urban slums in Kampala- Uganda, included; poor access to health services, limited knowledge about TB, TB stigma, language barrier and lack of facilitation of community health workers. Identified intervention strategies included; education, training, enablement, environmental restructuring and persuasion. The findings could serve as a guide for the design and implementation of interventions for improving the same.

**Supplementary Information:**

The online version contains supplementary material available at 10.1186/s12879-022-07283-9.

## Background

Tuberculosis (TB) remains a challenge among migrants and refugees despite a steady decrease in TB incidence among the general population in the last decade worldwide [1, 2]. Thus, achieving the World Health Organization’s (WHO) End TB Strategy of reducing TB deaths by 90% and incidence by 80% by 2035 [3], will not be realized without addressing TB among migrants and refugees. An inter-regional WHO consultation on TB and migration, highlighted the importance of analyzing the implications of migration for TB epidemiology, clinical care and health systems challenges and to translate evidence into effective TB control strategies anchored in principles of equity, ethics, and human rights [4]. Of recent many western countries have experienced increased rates of TB infection, with the disease disproportionally affecting foreign-born populations from Africa, Asia and/or Latin America, where TB infection rates are much higher [5]. Furthermore, recent evidence shows the spread of multi-drug resistant drug TB (MDR-TB) in East Africa as a result of refugees from Somalia- a high MDR-TB prevalent country, calling for urgent identification and management of cases for the countries in the region for humanitarian purposes and protection of their own residents [6].

Evidence shows that social and economic inequalities sustain migrants’/refugees’ vulnerability to TB. In addition, the unavailability of drugs, constant migration and breakdown of the infrastructure, pose a potential outbreak of TB and MDR-TB a serious threat [6]. The breakdown of the healthcare system and refugees leaving their countries of origin, they carry with them their TB across the region, thus causing serious setback in the fight against TB in the host countries, but also in where they transit [6]. Thus, absence of targeted TB prevention and control strategies for migrants/refugees create significant barriers in reaching TB elimination targets in several countries of origin, transit and destination for migrants [7]. Relatedly, addressing TB in migrants/refugees requires efforts on several fronts: cross-border collaboration, domestic strategies to optimize the diagnosis, treatment and prevention of TB, and minimization of the risk of TB transmission in migrants/refugees through support for TB care and prevention in high-burden countries [4].

Uganda, is one of the largest refugee-hosting nations in the world, with over 1.4 million refugees especially from South Sudan in the north, from Somalia in the north-eastern and from the Democratic Republic of Congo along the south-western borders [8]. Despite not directly bordering Burundi, Uganda received on average 30 Burundi refugees per day in September 2016, or 937 per month. The influx from the Democratic Republic of Congo (DR Congo) to Uganda has been continuous since 2014. In addition, following the armed conflict and turmoil in Somalia, the public health systems were disrupted causing patients to seek care in the neighboring countries [6]. Somalia and DR Congo are among top 20 high MDR-TB countries [9]. MDR-TB was estimated in 8.7% of new TB cases and in 47.0% of previously-treated TB cases in Somalia [10]. This differs much from the one in Uganda which is as low as 1.1% in new cases and 12% among retreatment [11]. The influx of large numbers of refugees places a strain on healthcare services in Uganda. In addition, there is no targeted TB prevention and control strategy for refugees and migrant populations which poses a big risk to the natives and also creates significant barriers in reaching TB elimination targets [3]. There is therefore a need to extend TB case detection and retention in care among migrant/refugee community, particularly those living in the urban slums due to increased risk of getting TB [12]. This study explored the barriers to and facilitators for TB case finding and retention in care and suggestions on how to foster TB case finding and retention in care among slum refugees in Kampala City, Uganda.

## Methods

### Study design, population and setting

**Fig. 1:**
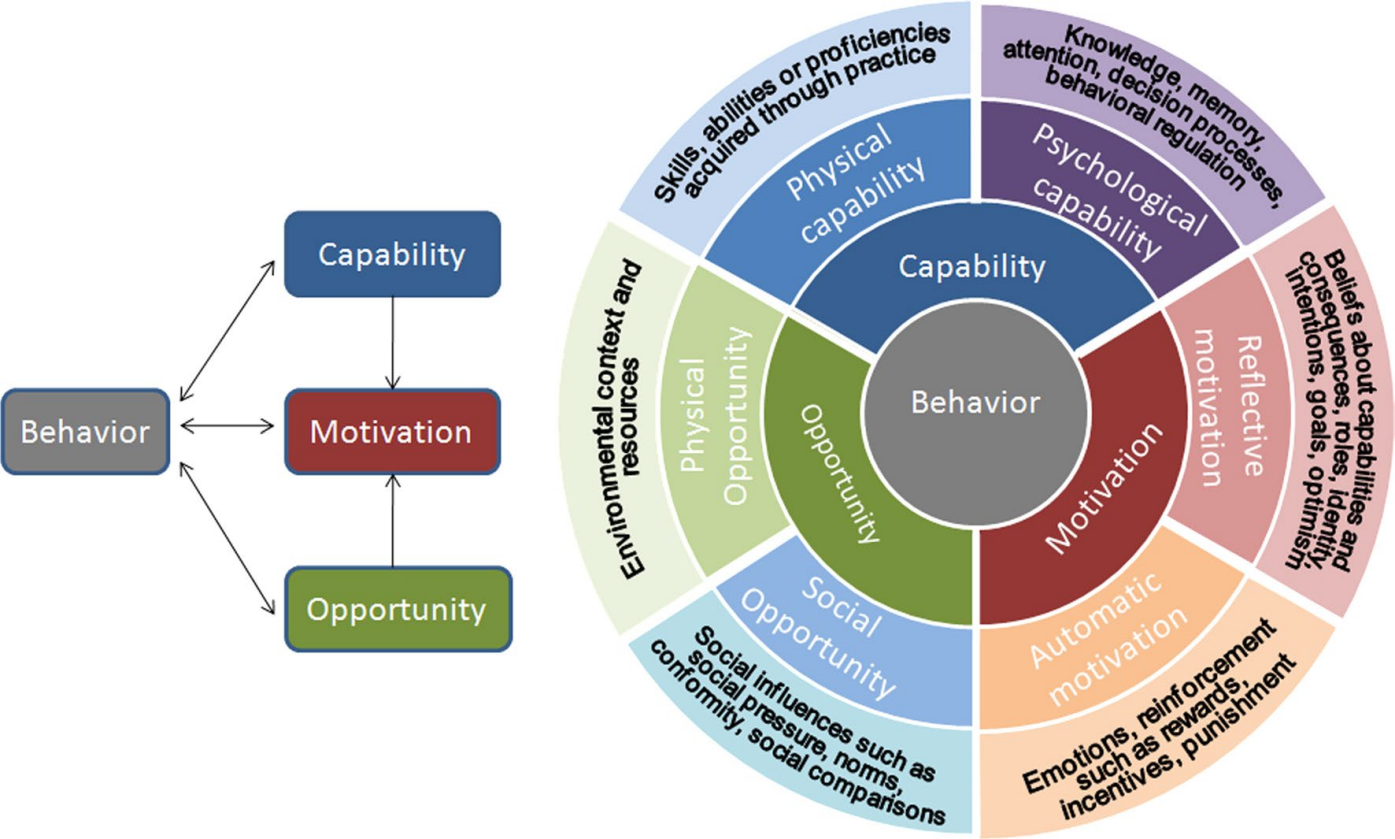
COM-B Model [13]

This was a cross-sectional qualitative study. In-depth interviews (IDIs) and key informant interviews (KIs) were conducted with front-line health care workers (including community linkage facilitators (CLFs) (also referred to community health workers), refugee TB patients and their care takers, community leaders and representative of implementing partner working with refugees in the urban areas. The study was guided by the Capability, Opportunity and Motivation Model of Behaviour (COM-B) model in Fig. 1 [13]. We then selected potential intervention strategies using the Behavior Change Wheel framework (BCW) [13], with the aim of improving TB case finding and retention in care among urban slum refugees.

The study was conducted in Kisenyi, the biggest slum in Kampala City. The city has approximately 1.5 million people and contributes 20% of the 47,000 TB cases notified in the country annually. Kampala has had a low TB performance due to health system constraints, mobile population, limited access to TB treatment, private sector engagement and weak social support system. With an estimated population of 20,000, Kisenyi slum is home to over 80% of all Somalis refugees coming from a high MDR-TB prevalent country [6], as well as other refugees from DR Congo, Rwanda and Eritrea. The living conditions in Kisenyi-slum consist of poor housing, overcrowding and poverty- perfect conditions for TB transmission including potential epidemic outbreak. Thus, the reason for selecting this study area.

### Data collection

*Participants* Data were collected between December 2019 and January 2020. KIs were conducted with, a representative of implementing partner working with refugees and front-line health care workers, including those involved in provision of TB services (diagnosis and treatment) at the government health facility and CLFs. The CLFs are attached to health facilities and help in conducting TB outreaches for TB, health education, case finding, contact tracing, and following up of TB patients in the community in case of loss to follow-up. They work as volunteers with TB projects, linking patients to the health facilities. IDIs were conducted with TB patients and their family members including care takers as well as community members and community leaders. TB patients were selected with the help of health workers and included males and females; drug susceptible/drug resistant TB patients (who had completed or still on treatment).

*Data collection tools* Data was collected using KI and IDI guides developed based on barriers to and facilitators for TB case finding and retention in care, guided by the COM-B model [13]. According to the model, behaviour is a result of an interaction between capability, opportunity, and motivation. Capability can be psychological (knowledge) or physical (skills), opportunity can be social (societal influences) or physical (environmental resources) while motivation can be automatic (emotion) or reflective (beliefs, intentions) [13]. The research team discussed the issues/topics to be explored by the interview guides (KI and IDI guides). The topics included; the patients’ experiences during TB diagnosis and treatment process, facilitators and barriers to TB diagnosis and retention in care. We also elicited respondents’ views on how best to improve TB case finding and retention in care for this population. Semi-structured guides were developed with follow-on questions or probes were used accordingly (Additional file 1).

Research assistants [2] with experience in qualitative research conducted the interviews. The research assistants received pre-training on the study objectives, research ethics and how to administer the interview guides. Each interview was audio-recorded, after getting informed written consent from the participant. Where the respondents couldn’t speak English or any of the local languages, translators facilitated interviews in Somali or Swahili.

### Data management and analysis

All audio-recorded interviews were transcribed verbatim by the research team. All transcripts were printed and copies reviewed independently by three authors (EB, EA and a LA) and manual content analysis was conducted after reading through the transcripts several times. Data was categorized by the constructs of the COM-B model [13]. In case of disagreement, consensus was sought through discussion by the three authors (EB, EA and LA). We used the BCW framework [13] to identify targeted interventions that are likely to be appropriate and effective in addressing the barriers to TB case finding and retention among urban slum refugees in Kampala City.

### Ethical considerations

The study was approved by the Makerere University School of Public Health Higher Degrees Research and Ethics committee (Protocol No. 705 (2019). All participants provided a written consent after receiving a thorough explanation of the study objectives.

## Results

### Characteristics of study participants

A total of 30 interviews was conducted and this was determined by saturation i.e. when no more new information came up. These included; 17 IDIs with patients (n = 10), 7 with patients’ caretakers/family members and 13 KIIs; eight with health care workers, one with implementing partner representatives, four local leaders (refugees and locals). The interviews with patients were conducted with both drug susceptible and drug resistant TB (who had completed or still on treatment), while the health workers included, four CLFs and four facility-based health workers at the Kisenyi HCIV (at a sub-district level serving about 100,000 people). The age of the respondents ranged from 18 to 45 years.

The findings are organized according to the tenets of the COM-B model categorized under barriers, facilitators, suggested solutions and identified intervention strategies. The barriers and facilitators to TB case finding and retention in care are integrated because there are majorly cross-cutting. Where necessary the similarities or differences, are highlighted. Illustrative quotes from respondents are used to exemplify the results.

## Barriers to TB case finding and retention in care

### Psychological capability

#### Limited awareness about TB

All respondents pointed out the lack of awareness about TB being common among refugees. This lack of knowledge about TB was felt to compromise proper health care seeking behavior, both prior to TB diagnosis and during treatment with severe consequences including transmission among households. Participants also highlighted poor understanding of TB symptoms with myths linking the disease to witchcrafts.

“Most of them don’t have information about TB. There is one who was treating it as a simple cough for a long time and all the four children and the parent got infected with TB. There is also one I worked on last week, he was saying ‘I don’t know what is causing me to sweat at night.’ So, they don’t know. Some say they are bewitched. Some say they are losing weight, when you mention TB some of them deny so they have little idea about TB.” TB Service Provider

The lack of knowledge reportedly created fear about TB. Participants said that TB was not discussed in communities to the extent that most people did not know that the disease is curable. As a result, TB patients were stigmatized and discriminated leading to isolation especially among people of Somali origin.

‘People in that community don’t have much information about TB, especially Somali people don’t know much about that disease... people don’t normally discuss about TB in the community. And that is the reason people fear TB so much. If they knew they wouldn’t fear it so much because it is just six months of treatment.’ Former Female TB patient

Some TB patients and care takers reported that they lacked information about where they could access TB services. They mentioned that they thought the drug shops and private clinics within their community were the only care providers, although only accessible to people with some money to pay. Most patients and care takers didn’t know about the free TB services available at the public health facilities

“I didn’t know here [referring to Kisenyi HCIV-public health facility]. When they sent me here, I even wondered that there was a hospital, because in the community we only have drug shops and when I went to those drug shops they told me it was only infection [meaning simple bacterial infection] and malaria, they did not tell me it was TB.” Former Male TB patient

This lack of awareness was also said to hamper TB outreach activities in the community. Health workers reported that because of the high burden of TB among urban slums, some TB programs organized TB outreaches in communities where refugees live. Although, the outreaches are meant to create awareness about TB as well conduct active case finding in the community including follow-up of TB patients who default on their treatment, it was reported that refugees usually shy away from such activities because of lack of awareness about TB disease.

“Before I was diagnosed with TB, I could not allow anyone to come to the community to test me just because I did not have any information about it but right now, I can test even five times.” Female Somali TB patient

### Physical capability

The barriers in terms of physical capability were expressed in terms of; inadequate TB services, lack of targeted TB prevention and control strategies for refugees and poor facilitation of health workers.

### Inadequate TB services

Services were described as inadequate and inaccessible. There is one government health facility-Kisenyi HCIV located in Kisenyi slum serving the refugees and the general population. This facility was reported to be having few health workers and overwhelmed with patients, affecting the quality of service. Besides the long lines at the only public health facility, some refugee respondents reported that they felt uncomfortable and unwelcome.

“Of course, when you come here [referring to Kisenyi HCIV] you line up for so long, so at times people fear to come here because when you come you take long in the line and you will stay here for long hours and when you are a Somali they don’t give you attention*.*” Female Somali TB patient care taker

Some refugee respondents felt that the facility was far from where they live compared to the drug shops and private clinics in their communities which most of them knew and found very convenient, especially for those who are able to pay.

However, besides being only profit driven, the private facilities were reported not to have adequate capacity to properly diagnose and treat TB because they are run by un-qualified staff. For example, it was reported that they treated patients who presented with TB symptoms for only malaria and simple cough, leading to delayed TB diagnosis.

*“*Before she was diagnosed with TB, my sister went to clinics and they checked and told her she had malaria. They treated her malaria for long, unfortunately malaria didn’t cure, and she had to come here [meaning Kisenyi HCIV]. When they checked they told her she had TB and she was put on anti TB*.*” Rwandan community health worker volunteer and former TB patient.

“When I went to clinics I didn’t get better. I could go to the clinic, buy medicines, but I continued to get fever, shaking, cough. So that is the reason I decided to stop going to clinics and went to that hospital. I went to clinics for three months. I took medicine for two months but I didn’t get any change*.”* Former Male TB patient.

### Lack of targeted TB prevention and control strategies for refugees

Key informants pointed out the lack of ‘targeted TB case finding and retention in care strategies’ specific for refugees as a barrier for TB control among the urban slum refugee community. It was reported that there was no specific strategy of TB screening of refugees as they enter Uganda and follow-up strategies for those living in slums in the country were lacking. Compared to refugee camps in Uganda with targeted TB services including periodic TB screening of people, the situation in the slums was different and the urban refugees were expected to utilize the same programs targeting the general population. However, these general programs were reported to be shunned by some refugees.

“I have not seen any system here that conducts TB screening among refugees. But in camps like Nakivale refugee camp, screening is regularly done. It would be a good idea [referring to TB screening], but it may not be possible like here where everyone [refugee] is on his/her own. Mobilizing them might not be easy, but in the camp people are organized and they are easy to get through the leaders in the camp*.*” Female Somali community member

### Lack of facilitation for health workers

Health workers pointed out that lack of facilitation for them including CLFs as one of the barriers for conducting community TB outreaches including contact tracing and follow-up of patients that are lost to follow-up. They reported challenges of poor motivation and facilitation such as lack of allowances, transport and supplies such as sputum mugs, protective gear (masks and N-95 respirators) to be able to carry out their work properly.

“…all in all, we don’t have facilitation, you know you cannot just walk to the communities. You need to be facilitated with some allowance as well as supplies to use. Their homes are poorly ventilated and visiting them is risking yourself. You need masks-N95 to protect yourself, especially when dealing with MDR-TB patients*.*” CLF3

### Social opportunity

The barriers under the social opportunity category, were described in terms of stigma and discrimination, language barrier, avoidance and uncooperative behavior; lack of collaboration with local leaders and across refugee camps.

### Stigma and discrimination

All respondents pointed out that the stigma and discrimination attached to TB was a big barrier to access and utilization of TB services including case finding as well as retention in care among the urban slum refugees. It was reported that the family members as well friends and neighbors reject TB patients when they get to know their status, to the extent that married women can be divorced by their husbands.

‘When they realize that you have TB in the community, they isolation you. And if you are a woman even your husband can divorce you.’ KI Female CLF2

“For them [Somalis] they say that if you test me and I am diagnosed with TB, the people I stay with are going to chase me from the house*…*” KI Male CLF3

Respondents reported that people run away from TB patients for fear of contracting the disease too. This stigma and discrimination attached to TB was mentioned to affect health seeking behavior. This was reported especially among Somalis who were described to always being impatient when visiting the public health facility, wanting to be prioritized over other patients who came before them. Some respondents felt that the impatience exhibited at the health facility by some refugees was to avoid being seen by their community members and labelled to be sick. This was especially complicated with implementation of TB control measures, such as wearing of a face mask which was felt to attract stigma.

“They [Somalis] come always in a hurry, wanting to be given priority before others. They don’t want to be seen at the health facility, because they will be discriminated. For example, “a patient who was diagnosed with TB and HIV from a private clinic was brought here [Kisenyi HCIV-public facility] for treatment. When I started health educating her, that was enough to irritate her, she removed the mask, she threw it away, and left. She escaped and went back to a private facility and that she said I am delaying her. I am exposing her to other Somalis to know that she is sick.*”* KI Health Care Worker

Also, the stigma attached to TB was felt to be partly the reason why some refugees preferred seeking care in the private health facilities. Refugees were said to feel more comfortable in private facilities where there are no long waiting lines as well as no wearing of masks. Wearing of masks in particular was felt to be stigmatizing as it labels the wearer to be sick. In addition, because of stigma TB patients feared disclosing their status to neighbors which affected how they accessed and used the health facility. One TB patient caretaker mentioned that whenever she is taking her nephew to the TB clinic, she lies to the neighbors that they are going to visit, in order to avoid being stigmatized and discriminated against.

‘Most of them don’t know that the boy has TB and when we are going to the health facility we pretend as if we are going somewhere to visit*.*’ Female Somali TB patient care taker

Similarly, other patients did not disclose to their own family members in fear of being rejected. For example, a young man of 21 years was diagnosed with TB, started treatment and completed without telling his parents. Another TB patient, who was also diabetic and on TB treatment shared his experience.

‘Even right now I am taking medicine but I did not tell people that I have it because besides TB I am diabetic…. Because I fear that people will start isolating me and they will start talking about me and they will spread the information in the community that I have TB and people will discriminate me…Because of this, I did not disclose to my family members. None of my family members knows that I have TB, they might also discriminate me*.’* Male TB Patient

This stigma and discrimination was also reported to affect participation in TB outreach activities, contact tracing and adherence to treatment as the refugees, particularly those of the Somali origin were reported not to allow health workers access their homes. Index TB patients were reported to give wrong home addresses, wrong telephone contacts or switching off their phones if they know a health worker was coming for a home visit, in order to avoid being followed up in their homes. This was felt to be because of fear of having their TB status inadvertently disclosed to the family members and the neighbors. One former TB patient shared his experience where health workers came home to do contact tracing when his sister-the index case in the family had TB and everyone in the house and neighborhood ran away.

‘Yes, they did it [meaning making a home visit to do contact tracing] but it was kind of tag of war. They had other people to convince us but it was not easy for them. Some people [within the household] even ran away after they were informed that health workers will be visiting to screen for TB. They could not stay and gave excuses for not being around.’ CLF1-Somali refugee and former TB patient

### Language barrier

All respondents mentioned that refugees, mainly from Somalia can’t communicate in the locally used languages including Swahili and English which compromised their health seeking behavior. Language difficulties and cultural differences were reported to be big challenges affecting both TB case finding and retention in care**.** Though some efforts were reported to be in place to solve the problem of language barrier by providing translators, this was still reported to be a problem as the translation services were not always available when needed.

*‘*It is very difficult for those who don’t know English or any other local language to communicate with the health workers. … People are many who don’t understand the language and they get challenges with accessing health care services in the community.’ Female Somali TB patient care taker

The challenge of language barrier was reported to be one of the reasons for making refugee process of seeking health care complicated and leading to patients’ delay at the health facility, particularly at Kisenyi HCIV, as health workers try to use signs to communicate as well as look for translators. There were also reports of difficult in finding appropriate translators to the extent that patients tried themselves to find translators from the roadsides. Other times, other patients are used as translators which may raise privacy concerns.

**“**Sometimes time is extended because of the language barrier, you need to go around and look for an interpreter if you don’t know the language to try and tell someone that now in this bottle we need sputum. Sometimes we use signs, when it comes to time for telling them that you are going to test for HIV. So in most cases the time you spend on someone increases if that person doesn’t know English and you don’t also know Swahili. Sometimes we use patients those who know Swahili and those who know maybe Congolese*.”* KI Health care worker

Some respondents further shared that language barrier was contributing to the delay in deciding to seek healthcare for sickness including TB among refugees who are not able to communicate in the locally used languages.

*‘‘…*some Somalis don’t know English and Swahili so some fear and end up delaying to come to the health facility because of language barrier.” KI Health Care worker

### Avoidant and uncooperative behaviour

Health workers and local leaders reported that refugees demonstrated uncooperative and avoidant behavior towards TB case finding and case retention activities. In connection with stigma, refugees were reported to display various forms of avoidant behaviour such an; not allowing health workers and local leaders visit their homes, giving wrong home addresses and telephone numbers when asked to give this information as is required from every patient to foster contact tracing and any other follow-up for the TB patients as may be required. The Somali community in particular was reported to be ‘very closed in’ and suspicious of outsiders. Both health workers and the local leaders mentioned how the Somalis deny access to their homes or have TB patients either hidden in communities and this was reported to affect contact tracing as well follow-up of any patient with a problem, in addition to implementation of other health interventions. But also, this avoidant behaviour was partly attributed to refugees committing crimes such as hosting fellow refugees who are illegally in the community/country. This was reported to also cause denial of health workers to visit their homes. It was further explained that being illegal in the community means the person has stay indoors and this was pointed out to affect health seeking behaviour, in case that person falls ill.

### Lack of collaboration and cooperation with family members, local leaders and across refugee camps

Health workers, particularly CLFs reported lack of support from the local leaders in helping mobilize refugees for outreach activities and also in helping trace TB patients during follow-up or contact tracing. The leaders were reported to ask for incentives in order to help health workers do their work. Further, the lack of collaboration across refugee camps in the country and across borders in terms of TB control was also alluded to be a barrier to tracking TB patients and retaining them in care.

‘When a client decides to go, you have no idea on how to follow-up him/her. You can’t know he/she has gone to a certain camp or back to his country of origin. You don’t even have or know whom to call and find out. Maybe if there was a leader of Congolese or Somalis in there that is the one you would call that there is so and so I don’t know whether he/she has reached there but we don’t have that contact.’ KI TB Clinic In-charge

## Physical opportunity

The barrier under the category of physical opportunity was majorly described in terms of poor living conditions and refugees being mobile.

### Poor living conditions

Refugees were reported to stay in poor living conditions, characterized by; high level of crowding and unhygienic environment. This was pointed out to be partly the reason why they don’t allow health workers and the local leaders to visit their homes.

### Refugees are mobile

One of the reasons for not adhering to the TB treatment was that the refugees, especially those of Congolese origin are very mobile and keep moving in between camps without proper transfer of the patient in terms of treatment continuation, thus compromising retention in care.

‘…another challenge is that because these people are refugees, we find difficulties in tracing them. Sometimes retention in care is hard because many of them will choose to go to other camps or back to their mother countries without completing treatment.’ Female CLF4

### Automatic motivation

The barriers under here were mainly expressed in terms of fear to be tested for TB and HIV and the stigma associated with a positive result.

### Fear of discrimination and rejection

Some respondents mentioned that refugees, particularly the Somalis because of fear of discrimination and rejection they don’t disclose their TB status, thus compromising contact tracing as well as adherence to treatment, in addition to affecting proper health seeking behaviour when they get TB symptoms.

“Somalis their problem is that they don’t want any other Somali to know that they are taking TB drugs… That they will be rejected and chased away from home.” KI Health Care Workers

Fear to disclose and don’t like to participate in contact tracing (most patients were against contact tracing).

‘I would not give attention to these people because remember I had not told anybody that I had TB and now how can they see people come following you up in the community…. People have stigma, they don’t want to be seen in public that they have TB because as I said when you are known to have it in the community the whole community will isolate you and this is because people don’t have knowledge about TB*.*’ Former male Somali TB patient

Because of the consequences, some respondents reported that refugees don’t adhere to the TB treatment due to fear to be known to have TB.

“Of course, if you are fearing because you don’t want others to know that you are taking TB treatment then you will not complete*..*.” KI clinic in charge

### Fear to be tested for HIV and TB

In addition to the fear of being stigmatized and discriminated against after being known to have TB as elaborated above under social opportunity, the fear of being tested for HIV was also pointed out to affect TB health seeking behaviour. Some people presenting with cough were reported to deny being evaluated for TB and HIV and would only want to be given only treatment for a simple cough.

“You will see someone coughing but when you ask them for sputum they don’t want to give it and they will tell you for me I just want to give me drugs for coughing, and because it is a requirement here for TB patients to test blood for them they don’t want to test them for HIV, but he will say I told you I am coughing why do you want blood again” CLF3

### Reflective motivation

#### Fear of long waiting time the public health facilities

The fear of the long patient lines in the public health facility was report to deter some refugees from seeking care there. As described above, some refugee patients are always impatient when vising the public health facility, partly because they are worried that their fellow refugees will get know that they are sick. In addition, the struggle they go through to communicate with the health workers, when they don’t know English or other local languages. But also, the implement ion of infection control measures, such as face masking scares away refugees from seeking care in the public health facilities.

#### Lack of facilitation for health workers and working under risky conditions

Lack of facilitation for the health workers, particularly the CLFs to conduct contact tracing and other outreaches in the community in an effort to improve TB case finding were also emphasized. Health workers in addition to lacking facilitation do outreaches, they operate under risky conditions by going to refugees’ homes in slums characterized by smoking and other drug misuse such as cocaine.

“We risk our lives, remember we go to areas where people take drugs, those people take cocaine, …someone can even rape you, and even these home visits we do are very dangerous, … you will enter there but you find a room smelling, someone maybe is smoking and the room is closed and there are no windows and you remember TB is air bone so we just survive by God’s mercy*.*” CLF4

### Side effects of TB medicine

Some patients were reported to abandon their TB medication because they experience side effects, thus being lost of in care and consequently dying. The side effects were reported to be more common and worse with drug resistant TB treatment. Such challenge was related to the many number of tablets to be taken for many months before completing the dose which discourages some patients with some of them deciding to rather die.

“Some [TB patients] when they are given drugs, they don’t want to take them. Some are given injections for many months. But even tablets become hard for people to take on a daily basis. This leads to some people to stop taking drugs and they opt to die. The drugs make it hard for people to swallow every day*.*” KI Local Leader

## Facilitators to TB case finding and retention in care

### Physical capability

The facilitators under the physical capability included; availability of free diagnostic and treatment services in the public health facilities, friendly health care workers, community outreaches and presence of CLFs.

### Availability of free diagnostic and treatment services in the public health facilities

Some respondents pointed out the availability of free diagnostic and treatment services- including free TB drugs in the public health facilities as a facilitator to TB case finding and retention in care in this population. In addition, easy access to the one public health facility within the Kisenyi community, in terms of less travel distance and related costs was also reported to facilitate both TB case finding and retention in care for TB patients in this slum community.

### Friendly and helpful health workers

Some patients reported that they found the health workers at Kisenyi HCIV in Kisenyi refugee community friendly and this motivated them to continue with their TB treatment.

“The health workers here are friendly and they are willing to help and I did not find any challenge here.” Male Congolese TB Patient on treatment

### Community outreaches and presence of community linkage facilitators

Health workers reported that outreaches in the community to foster TB case finding in the community were organized by some projects/implementing partners, targeting both the refugees and the locals. In addition, home visits were mentioned to be conducted in order to do contact tracing as well follow-up patients who have been lost to follow-up. These were reported to be conducted by the CLFs, who play a very critical role in linking the communities to the health facilities, as well as conducting awareness campaigns. Although, some refugees were non-responsive in such community activities, CLFs were felt to be supportive in providing opportunities for active case finding, where health services are brought to the community to try identify TB patients earlier than waiting for the patients to bring themselves to the health facilities, which usually happens at an advanced stage of the disease.

“We go out and talk to people and when we put a camp some people come and we sensitize them. They get to know and when they experience TB symptoms they know where to go. Instead of someone saying I cough blood when they don’t know why they are coughing blood. At times whenever we go to the community we leave our phone contacts with them when they get any problem they call us. ‘That come here we have this person who is suspected to have TB” CLF3

This concept was also highlighted under the facilitators falling under Psychological capability. In addition, past experience with TB disease was reported to result in being aware of the TB disease.

### Social opportunity

The presence of translators; family support and working with refugee local leaders were the major themes under social opportunity.

### Presence of translators

Some respondents (both patients and health workers) reported that the presence of translators were useful in fostering communication between the patients and health workers addressing the challenge of language barrier. This was pointed out to facilitate communication with health workers during TB diagnosis and retention in care of the refugee TB patients. Some translators were reported to be provided by the implementing partners working with refugees.

### Family support

Support from family members was also raised as a facilitator to retention of care among refugee TB patients. Though disclosure was generally felt to be a problem due to TB stigma, with patients even not disclosing to their family members, a few patients who reported they had disclosed to some of their family members, confessed being supported to adhere to treatment.

‘At the beginning I got scared and I had thought of stopping taking the medicine but slowly with time I got strong and continued with my treatment. The period of medication was too long and the tablets were too many. Every time you take them you feel like vomiting. That is when I felt like stopping, but my mother kept encouraging me.’ Former Somali female TB patient

### Working with refugee local leaders

Working with the refugee leaders in organizing the health activities that foster TB case finding and retention was pointed out to help in getting the refugees participate. Because of their influence and importance in their communities, the refuge community leaders were supportive in mobilization for a number of TB control activities including outreach camps.

“It is noted that activities we have done with organization have come out very well that what we have tried as individuals, like we had Congolese in Makindye we had tried them ourselves and we had failed to get them. But when we went through their leader and the organization contacted them they came out and we got a good number of them so bringing these people [refugee leaders] on board is good.” CLF3

## Suggested interventions

### Psychological capability

#### Creating awareness for TB

All the respondents expressed the urgent need to create awareness about TB among the refugees. Creating awareness on causes, symptoms and prevention of TB was mentioned to be very critical as it would address many issues including changing attitude, myths about the disease and dealing with stigma which was felt to be a big barrier, but also to help improve health seeking behaviour for TB.

‘You need to health educate people in the community who don’t know about TB so that they can get to know what it is and how you can prevent it and how you can support someone who has it, then reduce the stigma in the community.’ Female TB Patient

Suggested strategies to use for creating awareness include using social gathering like schools and mosques, as well as social media in addition to organizing health activities such as outreach camps specific for refugees.

### Social opportunity

The social opportunities were presented in terms of implementing partners integrating TB services in their work, and working with refugee leaders, and increasing the translators.

### Implementing partner integrating TB control services

Some respondents pointed out that it would be easier for the implementing partners working with refugees to integrate TB services including case finding and retention interventions in all their services for refugees given that they closely work with them. This can include such partners establishing proper registers of all refugees to ease follow up and tracing.

### Working with refugee leaders

The idea of health facilities working in collaboration with key stakeholders including influential people in communities such as local leaders (refugee community leaders) and religious leaders was mentioned to help implement all the activities related to TB control among refugees.

“The health facilities should work with either NGOs or the leaders of the refugees, then after working with them, we will put camps. Of course, now when we are working with them it will be their leaders who will go and call them come to be screened for TB” KI clinic in charge.

### Availability of translators

*G*iven that most refugees were not able to communicate in the locally used languages, it was felt that the implementing partners should have translators always stationed at the health facilities, so that they can be easily accessed whenever needed.

### Reflective motivation

#### Integrate TB services with non-stigmatizing diseases

It was also suggested that in order to deal with the stigma attached to TB, TB services such as outreaches including contact tracing and follow-up of TB patients, should be integrated with other non-stigmatizing diseases such as hypertension, diabetes, malaria. Participants felt that this would enhance privacy with a health worker, during which the person can be informed of an opportunity to screen for TB as well.

“Some people come out and tell you that me I feared to ask you in public [meaning in an outreach camp, but I am like this and this. And when you test the person you will find the person with TB.” CLF3

#### Facilitation of health workers

Facilitation of health workers particularly the CLFs who conduct outreaches in the community was pointed out to be important. This facilitation should include transport, their allowance, provision of personal protective equipment and other necessary supplies in order to carry out their work well. Motivation was mentioned not only for health workers, but also the local leaders in the community. This would support different TB control strategies including case finding, community entry and observing TB treatment.

A summary of the barriers for TB case finding and retention in care among urban slum refugees in Kampala, Uganda, Table 1 and identified intervention functions in Table 2.

**Table 1:** Barriers to TB case finding and retention in care and identified intervention strategies based on COM-B model and BCW framework among slum refugees in Kampala, Uganda

Behavioral determinant	TB case finding and retention	Suggested interventions	BCW intervention strategies
Barriers			
a) Capability			
Psychological	Limited awareness about TB	Creating awareness for TB	Education
Physical	Inadequate TB services Lack of targeted TB prevention and control strategies for refugees Lack of facilitation for health workers		Training Enablement Environmental restructuring
b) Opportunity			
Social	Stigma and discrimination Language barrier Avoidant and uncooperative behaviour Lack of collaboration and cooperation with family members, local leaders and across refugee camps	Implementing partner integrating TB control services Working with refugee leaders Availability of translators	Education Persuasion Enablement Environmental restructuring
Physical	Poor living conditions Refugees are mobile		Education Enablement
c) Motivation			
Automatic	Fear of discrimination and rejection Fear to be tested for HIV and TB		Education Enablement
Reflective	Fear of long waiting time in the public health facilities Lack of facilitation for health workers and working under risky conditions Side effects of TB medicine	Integrate TB services with non-stigmatizing diseases Facilitation of health workers	Enablement Environmental restructuring

**Table 2:** Identified intervention functions

Intervention functions	TB case finding and retention in care	Behavioural determinant
Education	Increasing knowledge and understanding of TB among the refugees to foster proper health seeking behaviour and reduce stigma	Psychological capability
Training	Training the private providers in TB diagnosis and management-linking them to public health facilities (public–private partnership) and incentivization of private clinics	Physical opportunity
Persuasion	Using communication to induce positive feels and dispel negative ones-using information educational materials (IEC), thus addressing TB stigma and fostering acceptability of TB services such as contact tracing and attending outreach services	Social opportunity and reflective motivation
Enablement	Facilitation for health care workers who follow-up patients in the community as well as doing community outreaches Integration of TB services with non-stigmatizing ones like HT, Diabetes Working with implementing partners, health facilities and local leaders to put in place regular screening for TB and other diseases specific for refugees	Physical opportunity and reflective motivation
Environmental restructuring	Integration of TB services with non-stigmatizing disease services such as hypertension and diabetes mellitus during outreach campaigns Promoting private–public partnership in TB diagnosis and retention in care Working with implementing partners, health facilities and local leaders to put in place regular screening for TB and other diseases specific for refugees	Physical opportunity

## Discussion

This study explored barriers to and facilitators for TB case finding and retention in care among the urban slum refugee population in Uganda. We identified multifaceted and interrelated facilitators and barriers to TB case finding and retention, as well as suggestions on how to better control TB in this population. Barriers to TB case finding and retention in care were mainly due to stigma, lack of TB awareness, inadequate TB services in the community where refugees mainly reside, access to ill-equipped private facilities with no capacity to diagnose and manage TB, and language barrier that negatively affects access to health services. In addition, poor facilitation of health workers who conduct TB outreach activities (for active case finding, including follow of TB patients and contact tracing) and refugees being uncooperative, with avoidant behaviors. Availability of free TB services in the public health facility and friendly health care workers were pointed out as facilitators for TB case finding and retention. Suggestions on how to improve TB control in this population were; TB health education to create awareness, making translation services always available and establishing collaboration between health facilities, the refugee local leaders and the implementing partners working with the refugee population. Furthermore, participants pointed out the importance of putting in place targeted TB control measures for the urban refugees as well as integration of TB services with non-stigmatizing disease services. Identified intervention strategies included; education, training, enablement, environmental restructuring and persuasion.

Stigma related TB was reported to be very common in this population, particularly among the Somali population. Stigma was reported to compromise the whole cascade of TB case finding to retention in care. With regard to TB case finding, refugees mainly those of Somali origin were reported not participate in the TB outreaches organized in the communities. These outreaches serve many purposes; creating awareness about TB as well for active case finding as the people with TB symptoms are supposed to approach the health workers and have them evaluated. For example, if one has cough, they are asked to provide sputum sample for laboratory diagnosis. Using this approach of outreaches/camps, is efficient in achieving early TB diagnosis and reducing TB transmission in the community. For those who go the health facilities were reported to always be impatient, mainly because of fear of being seen in the health facility, plus wearing of masks for TB infection control, meaning the person is sick and they will be stigmatized. Partly to avoid seen queuing at the health facility as well evading wearing of face masks in public health facilities, refugees were reported to prefer to seek care in the private health facilities-such as drug shops and private clinics where it is convenient and usually no need to que. Furthermore, stigma affected contact tracing, disclosure of patients TB status (even to family members) as well as affecting adherence to treatment. This is because TB patients once discovered, would be discriminated against including being isolated, or sent away from their homes. TB stigma has for long known to create a barrier to testing, treatment, and retention in care, particularly in low and middle-income countries [13]. It was also found to be a barrier to TB contact tracing in a study by Ayakaka and colleagues in an urban non-refugee population in Uganda, with index patients fearing loss of privacy and face discrimination from the household and community members [14]. Thus, stigma is one of the major barriers to achieving the TB End Strategy [3]. One of the interventions strategies identified was training/health education about TB. Health education/training about a condition or disease has been documented as one of the interventions in addressing stigma [15].

Language barrier was found to affect health seeking behaviour for TB in general. In the case of refugees, language barriers coupled with lack of translators at health facilities and in TB outreach events in the community impedes health care seeking. On the whole most refugees were reported not to know English and the other commonly used local languages, thus they could not participate in such activities. This is similar to what was found in a study by Chuah and colleagues in Malaysia, where language barriers and low literacy of the refugees and asylum-seekers, led to lack of understanding the health information relayed to them by health workers [16]. Because of this, they highly depended on their community leaders or members as information sources on how to navigate the health system and as translators when seeking medical care at public health facilities [16]. However, this seems not work for the Somali refugees because they are a very ‘closed in community’ and don’t even reach out for help from the community they live in including the local leaders (non-Somali) and feel everyone is their spy. This poses a great challenge in health promotion and prevention given that they tend to live in situations of overcrowding [17], making them more prone to diseases. Targeted interventions for these urban refugees are critical, in order to address such barriers and achieve TB control. However, this contravenes the policy in Uganda of integrating health services for refugees into the national health-response as a long-term solution [17]. Health services for refugees in Uganda are integrated with those of the host communities [8]. But also, refugee/migrant populations face many barriers in accessing essential services, especially illegal ones may choose to avoid public services due to distrust and fear of deportation, thus missing out on important promotive health measures [17].

One of the suggestions to improve the situation of TB control among refugee population in this study was working in collaboration with influential local leaders (Somali community leaders) and religious leaders of refugees and implementing partners working with the refugee population. But also, health worker and patient respondents also pointed out the role of implementing partners in mediating between refugees and the health facilities. This is similar to what was found in Malaysia by Chuah where refugee clinics run by implementing partners were perceived to play an important role in facilitating the navigation process through its clinical referrals that are accompanied with information on where and how to seek further healthcare services at government public health facilities as well as assist with translation [16]. Engaging refugee local leader is similar to what has been found elsewhere in Africa as community leaders are the gatekeepers of their communities [18]. The other suggestion to deal with the TB stigma, refugee respondents suggested that TB activities should be integrated with other diseases which are not stigmatizing e.g., hypertension, diabetes, that way people will be willing to participate. Integration of disease intervention in addition to dealing with stigma, it is also cost-effective [19]. The idea of having special TB control programs as well as working in collaboration with key stakeholders for refugees rather use the existing services for the native is related to what Lonnroth [4] pointed out that in order to address TB among migrants/refugees requires efforts on several fronts; including; collaboration, domestic strategies to optimize the diagnosis, treatment and prevention of TB [4].

Seeking care in private health facilities such as clinics was reported to be common among the refugees and also associated with delays in TB diagnosis, with consequences of transmission to close contacts of the TB patients. Consulting private health facilities has been shown to cause delay in TB diagnosis [20], including giving wrong diagnosis to the patient, thus delaying seeking further treatment [21]. This calls for training of HCWs in the private facilities in TB diagnosis and management as well as the implementation of public and private partnership. Our study had strengths, including the use of a behavioural framework for intervention design, the participation of health care workers, patients and their caretakers in exploring the barriers to and facilitators for TB diagnosis and retention in care among urban refugee population. Our study also had limitations. It was, a qualitative study which limits generalizability of our study findings. In addition, we captured perspectives of patients who had been diagnosed and were currently undergoing or had completed treatment and this may not fully capture the barriers experienced by people who avoided testing and treatment.

## Conclusions

This study highlights serious challenges faced by refugees in accessing TB services ranging from limited knowledge about TB, stigma towards TB and language barriers while seeking health care. TB disproportionately affects refugees/migrants, yet this population faces unique challenges in accessing quality health care as shown by the findings of this study. Further research needs to be undertaken, implementing the suggested solutions i.e., incentivization, training, enablement, and restructuring of the service environment as relevant intervention functions.

## Supplementary Information

**Additional file 1.** The tool that was used to conduct the interveiews.

## Data Availability

The data that support the findings of this study are available from the main author, however restrictions apply to the availability of these data, given that the study only utilized qualitative data. In general, it is difficult to fully de-identify qualitative data and difficult to interpret without understanding of the context. In addition, there were local terms (commonly spoken language) in the text. As a result, this makes it hard to share the source data. Data are however available from the authors upon reasonable request and with permission from the principal investigator.

## Abbreviations

CLFs: Community linkage facilitators
COMB-B: Capability, Opportunity and Motivation Model of Behaviour change Model
IDIs: In-depth Interviews
KIS: Key informant interviews
MDR-TB: Multi-drug resistant drug tuberculosis
TB: Tuberculosis
