# Supplementary material for: Developing strategies to address barriers for tuberculosis case finding and retention in care among refugees in slums in Kampala, Uganda: a qualitative study using the COM-B model
Source: BMC Infect Dis. 2022 Mar 28;22:301. doi: 10.1186/s12879-022-07283-9 (PMC8962141; doi:10.1186/s12879-022-07283-9)
Supplement: Supplementary file 1 — Additional file 1. The tool that was used to conduct the interveiews. [file 12879_2022_7283_MOESM1_ESM.docx]

### Study tools

**Interview guide for family members of TB patients and the community**

**Local Perception of TB as a disease**

**Interview guide for family members and community**

1. [Ice breaker] What are your views on health service delivery in your community?
2. How common is TB in your community/migrants?
3. Tell me about the local understanding of TB as a disease in this community? (probe for local names/terms used to describe the disease, perceived causes, modes of transmission, gender differences)
4. What does being infected with TB mean in this community/migrants? (probe for: society understanding of TB, Issues of witchcraft, ritual/spiritual issues, God’s plan, one has HIV/AIDS, weak blood group, death sentence in relation to TB)
5. Please explain why people have such view/s?

**Health seeking behavior prior to TB treatment**

1. Before starting TB treatment, what challenges do TB patients face in accessing care? (probe: knowledge and information about TB, social challenges, accessing to health facilities-health workers)
2. What support systems are there to foster TB diagnosis and retention in care for the migrant community? [Probe: social support-language translation, health system e.g VHTs, picking sputum specimens and delivery of results]. How successful or unsuccessful have these support systems been.
3. What do you think can be done to improve/fill these challenges you have mentioned?
4. Is there any follow-up for your family or household contacts? If yes, how was this handled? What are you views on the exercise of contact tracing? Probe: whether they liked or didn’t like the exercise
5. a) Are there arrangements for actively screening for TB among your community? If yes, how do they operate (probe for who does the active case finding, where is it done from? What exactly do they do during active case finding?)

b) If the process of actively looking for TB cases in your community is being done, do you have any concerns and challenges about it? If yes, what are you concerns? How best can it be done?

**Active case finding**

**According to WHO recommendation;** shows that refugees and migrants are more prone to developing TB and because of this, it is recommended that such groups of people should be actively screened/checked for TB. If found to have it, they should be started on treatment immediately in order to improve their recovery from the disease, but also to stop the chain of transmission to others in their homes and community.

.

1a) What do you think would be the community’s view regarding regularly screening/checking for TB among refugees/migrants?

b) What could be the barriers to this regular screening/checking for TB among refugees/migrants?

c) What could be the facilitators to this regular screening/checking for TB among refugees/migrants?

2. How can TB be best prevented/controlled in the community? (Probe for: what information messages, what services, barriers …)

3. What are the main sources of information on TB in the community? (probe for radio, peers, health facilities …)

**Experiences in treatment and care**

1. What treatment options are available for TB patients in your community? (probe for the most frequently used: traditional healers, herbalists, spiritualists, self-medication, formal health facilities …) For you, what treatment options are you on?

2. How do patients cope with treatment? Probe for: side effects, access to medicines, nutrition, social support-stigma, transport costs.

3. What are/were your support systems for TB treatment? [Probe: social support-, health system e.g VHTs, supervised treatment-DOT, transport to health facility, nutrition support, counseling, grant support, food or transport assistance, community health worker, social

work, etc.). How successful or unsuccessful have these support structures been/were? What can be improved.

4) What are your experiences at the clinic? (probe: how is your relationship with providers, what do you think about the quality of care you receive?)

6) What challenges have you faced and how have you dealt with them? (Probe for: Social issues-stigma; health systems-issues)

7) Do you have any preferences or suggestions going forward? (probe: if you could change something, what would it be?)

**Interview guide for health care workers** on TB control strategies among refugee/migrant population

Preamble: Refugees generally have an increased risk of getting TB- given the circmushigh prevalence of TB from Somali (including MDR-TB) because of the breakdown of the health system in the country. WHO recommends strategies for such high TB risk groups to be actively screened for TB, do contact tracing in order to identify TB cases early and start them on treatment to interrupt the chain of transmission.

1. How common is TB in this community? [probe for refugees/migrant’, Somali migrants]?
2. What challenges face TB diagnosis and retention in care for among refugees/migrants in this community?
3. What facilitators that foster TB diagnosis and retention in care for among refugees/migrants in this community?
4. What strategies are currently implemented to foster for TB diagnosis and retention in care for TB [patients among the refugees/migrant population in this community? [probe: Active case finding; contact tracing., cross-border collaboration, domestic strategies to optimize the diagnosis, treatment and prevention of TB in immigrants]. How successful or unsuccessful are those strategies?]
5. Does your health facility conduct active case finding for TB patients? If yes, is active case finding conducted among the Somali population? a) What challenges do you face in conducting active case finding in general population b) what are the challenges/facilataors you face related to active case finding among the refugee/Somali refugee population in Kisenyi?
6. WHO recommends contact TB case finding for all TB cases. Does your health facility conduct contact TB case finding for TB patients? If yes, is Probe for contact TB case among a) general population; refugee/Somali population. What challenges and facilitators do you face in conducting contact case finding this community?
7. How can an active case finding program best be implemented in the refugee population, how about the Somali migrants? Probe: a) facilitators and, b) barriers.
8. ) What suggestions do you have in terms of strategies to optimize diagnosis, treatment and retention in care for TB among refugees/ migrants from Somali and other areas? (Probe: cross-border collaboration, domestic strategies to optimize the diagnosis, treatment and prevention of TB in immigrants).
9. Suggest strategies to optimize the diagnosis, treatment and prevention of TB in migrants/refugees.
